# Sex‐Related Differences in Outcomes of Endovascular Treatment in Large Vessel Occlusion Stroke—Analyses From the German Stroke Registry‐Endovascular Treatment

**DOI:** 10.1111/ene.70092

**Published:** 2025-02-26

**Authors:** Constanze Single, Annerose Mengel, Kornelia Laichinger, Jennifer Sartor‐Pfeiffer, Nadja Selo, Florian Hennersdorf, Benjamin Bender, Milani Deb‐Chatterji, Götz Thomalla, Joshua Mbroh, Sven Poli, Ulf Ziemann, Ulrike Ernemann, Katharina Feil, German Stroke Registry—Endovascular Treatment (GSR)

**Affiliations:** ^1^ Department of Neurology & Stroke Eberhard‐Karls University Tübingen Germany; ^2^ Hertie Institute for Clinical Brain Research Eberhard‐Karls University Tübingen Germany; ^3^ Department of Neuroradiology Eberhard‐Karls University Tübingen Germany; ^4^ Department of Neurology University Medical Centre Schleswig‐Holstein Kiel Germany; ^5^ Department of Neurology University Medical Center Hamburg‐Eppendorf Hamburg Germany

**Keywords:** acute management, endovascular thrombectomy, female, intensive care, intravenous thrombolysis, sex‐related differences, stroke

## Abstract

**Background:**

Sex‐related differences in acute ischemic stroke may affect outcomes, yet evidence remains inconsistent. This large‐scale study investigated sex‐related differences in clinical presentation, peri‐interventional parameters, and outcomes after endovascular thrombectomy (EVT) for large vessel occlusion (LVO) using data from the German Stroke Registry—Endovascular Treatment (GSR‐ET).

**Methods:**

We analyzed 11.896 EVT patients (52.2% female) from the GSR‐ET (June 2015–December 2021) comparing clinical characteristics, treatment details, and outcomes by sex. Two propensity score matchings (PSM) were applied: (1) logistic regression model with a caliper width of 0.1 on age, pre‐stroke modified Rankin Scale (pmRS), and National Institutes of Health Stroke Scale at admission, and (2) 1:1 nearest neighbor matching with a caliper of 0.01. Primary outcomes were good (mRS 0–2) and excellent (mRS 0–1) outcomes at discharge and 90‐day follow‐up.

**Results:**

Women were older (76.3 ± 12.7 vs. 70.2 ± 12.9 years, *p* < 0.001) and had higher pre‐stroke disability (median pmRS 0 (0, 2) vs. 0 (0, 1), *p* < 0.001). Cardioembolic strokes were more frequent in women, even after PSM. Despite this, women had better odds of achieving good outcomes at discharge (adjusted OR 1.20, 95% CI 1.04–1.38, *p* = 0.013), but not at follow‐up (OR 0.91, 95% CI 0.78–1.05, *p* = 0.193). Both PSM analyses confirmed these findings.

**Conclusions:**

While women demonstrated better short‐term functional outcomes after EVT, these benefits diminished in follow‐up. The persistence of cardioembolic stroke in women suggests potential sex‐specific mechanisms. Understanding and addressing sex‐related differences in stroke is essential to optimize acute stroke care and improve outcomes. Future studies should explore biological and socio‐economic factors influencing sex‐related differences.

**Trial Registration:**

ClinicalTrials.gov identifier: NCT03356392

**Abbreviations**
(p)mRS: premorbid modified Rankin Scale
ACA: anterior cerebral artery
AE: adverse event
AF: atrial fibrillation
BA: basilary artery
CT: computed tomography
CTA: CT angiography
ECASS II/III: European Cooperative Acute Stroke Study part II/III
EVT: endovascular thrombectomy
FLR: flow restoration
GRO: groin puncture
GSR‐ET: German Stroke Registry‐Endovascular Treatment
ICA: internal carotid artery
ICH: intracerebral hemorrhage
IVT: intravenous thrombolysis
LSW: last seen well
LVO: large vessel occlusion
MCA: middle cerebral artery
mTICI: modified Thrombolysis in Cerebral Infarction score
NIHSS: National Institutes of Health Stroke Scale
PCA: posterior cerebral artery
PSM: propensity score matching
RCT: randomized controlled trials
rtPA: recombinant tissue plasminogen activator
SD: Standard deviation
sICH: symptomatic ICH
sICH: symptomatic intracranial hemorrhage
SMD: standardized mean differences
SO: symptom onset
VA: vertebral artery

## 1 Introduction

Growing evidence underscores the importance of sex‐related differences in acute ischemic stroke, with women not only exhibiting a higher lifetime prevalence and worse outcomes but also a diminished quality of life after stroke compared to men [1, 2, 3, 4]. Several major risk factors, including hypertension, smoking, and atrial fibrillation (AF) are more strongly associated with increased stroke incidence in women [5]. These differences may arise from unique physiological aspects in women, including coagulation pathways, immune response, and hormone exposure [6, 7]. Despite often presenting with more severe symptoms and residing at similar distances to medical facilities, women are less frequently directed to comprehensive stroke centers and exhibit delays from symptom onset to hospital arrival, largely due to delayed calls to emergency services [8, 9]. Once hospitalized, disparities in the utilization of acute reperfusion therapies may further exacerbate sex‐related differences in outcomes. Intravenous thrombolysis (IVT) and endovascular thrombectomy (EVT) for large vessel occlusion (LVO) have markedly improved stroke outcomes, effectively doubling the likelihood of favorable recovery in both anterior and posterior circulation stroke [10, 11, 12]. However, women are consistently underrepresented in these therapies. A meta‐analysis found women were 13% less likely to receive IVT [13]. Female sex was an independent predictor of worse 90‐day outcomes in lacunar stroke treated with IVT [14]. Women with LVO were less often selected for EVT, contributing to poorer outcomes [15, 16]. Although randomized controlled trials (RCT) generally reported negligible sex‐related differences in EVT efficacy [17, 18], observational studies and registry data often indicated worse outcomes for women [19, 20, 21, 22, 23]. These findings are often attributed to confounding factors such as older age and worse pre‐stroke functional status in women [24], highlighting the complexity of sex‐related differences in stroke outcomes. Beyond these, variations in clinical presentation, imaging characteristics, and periinterventional parameters may also play a role [25].

Our study aimed to address these gaps by comprehensively investigating sex‐related differences in clinical presentation, periinterventional parameters, and outcomes following EVT. Leveraging the prospectively collected data from the German Stroke Registry–Endovascular Treatment (GSR‐ET), we minimized potential biases by employing propensity score matching (PSM) to adjust for confounders.

## 2 Methods

### 2.1 Study Design and Ethics

**FIGURE 1:**
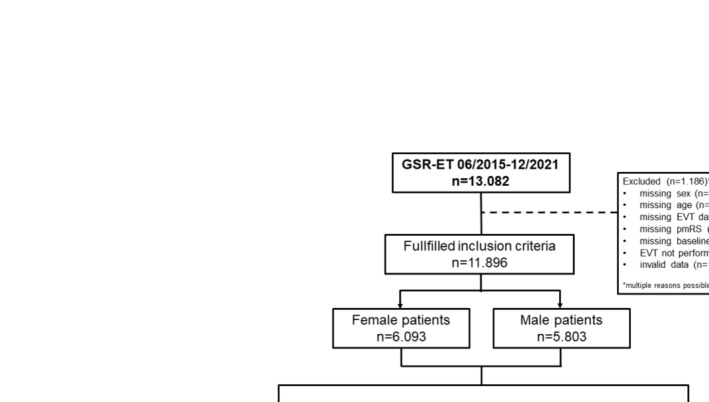
Flowchart of selection process. EVT, endovascular treatment; GSR‐ET, German Stroke Registry—Endovascular Treatment; *n*, number; NIHSS, National Institutes of Health Stroke Scale; pmRS, pre‐stroke modified Rankin Scale.

This retrospective observational study analyzed data from the GSR‐ET (https://www.clinicaltrials.gov; NCT03356392), an ongoing, open‐label, prospective, multicenter registry [26, 27]. Institutional review board approval was obtained from Ludwig Maximilian Universität, Munich, Germany (689‐15) and University of Tübingen (057/2016BO2) with informed consent provided. Local ethics approval were obtained according to site regulations. The analysis followed STROBE reporting guidelines (Supporting Information). Of 13.082 EVT patients in the GSR‐ET, *n* = 1.186 patients were excluded (Figure 1).

### 2.2 Definitions

Stroke severity was assessed using the National Institutes of Health Stroke Scale (NIHSS) and functional dependence using the modified Rankin Scale (mRS). Good outcome was defined as mRS 0–2 and excellent outcome as mRS 0–1 according to the ESO (European Stroke Organisation) definition (https://eso‐stroke.org/outcome‐measures‐stroke‐modified‐rankin‐scale‐ordinal‐logistic‐regression/). Substantial neurological improvement was defined as a difference between admission and discharge NIHSS of ≥ 8 points or discharge NIHSS of ≤ 1 [28, 29]. Reperfusion success was measured by the modified Thrombolysis in Cerebral Infarction (mTICI) score [30]; mTICI 2b–3 was rated as successful reperfusion. A modified CHA2DS2‐VASc Score estimated cardiovascular risk, adjusted for data variability including the following factors: age (≥ 75 years: 2 points, 65–74 years: 1 point), female sex (1 point), hypertension (1 point), diabetes mellitus (1 point) and previous stroke (2 points). Intracerebral hemorrhage (ICH) was defined as any hemorrhage in routine CT postinterventional imaging 24 h after EVT according to the European Cooperative Acute Stroke Study part II (ECASS II) [31], irrespective of the presence of new clinical symptoms, while symptomatic ICH (sICH) was defined as any ICH on follow‐up imaging leading to an increase in NIHSS of ≥ 4 points from baseline to 24 h‐value (ECASS III) or death [32].

### 2.3 Outcomes

Primary outcome was mRS at discharge and 90‐day follow‐up. Secondary outcomes included NIHSS at discharge, (peri‐)procedural time intervals, ICH and sICH, and periprocedural and in‐hospital complications.

### 2.4 Statistical Analysis

Continuous variables were tested for normal distribution using the Kolmogorov–Smirnov test. Normally distributed data were presented as mean and standard deviation (± SD), while non‐normally distributed data were presented as median with interquartile range (IQR) with 25th and 75th percentiles or counts and percentages. Clinical characteristics, imaging, periprocedural times, and outcomes were compared using the Kruskal–Wallis or median test. Continuous variables were assessed with the Mann–Whitney *U* test, and categorical data with the Chi‐square test. Binary logistic regression analysis evaluated good (mRS 0–2) and excellent (mRS 0–1) outcome, and mortality (mRS 6) at discharge and follow‐up, including significant univariate variables and known predictors. Model fit was assessed with the Hosmer–Lemeshow test, using a binomial distribution and logit function.

Propensity score matching (PSM) was performed using two approaches: The first PSM cohort used a logistic regression model to estimate the propensity scores based on age, pmRS, and NIHSS at admission, with a caliper width of 0.1 standard deviations of the logit of the propensity score, without replacement. The second PSM cohort employed 1:1 nearest neighbor matching without replacement, based on age, pmRS, NIHSS at admission, and comorbidities (hypertension, AF, dyslipidemia, diabetes mellitus, smoking). Missing categorical variables were imputed using a dummy variable to maintain data integrity. A caliper of 0.01 was employed. The effectiveness of the matching procedure was assessed by comparing the standardized mean differences (SMD) with a SMD < 0.1 being indicative of adequate balance. For the matched cohort, logistic or conditional regression analysis evaluated the association between gender and stroke outcomes, presenting adjusted odds ratios (aORs) with 95% confidence intervals (CIs).

Statistical analyses were conducted using SPSS Version 29.0.1.1 and R 4.3.2. The statistical significance level was set to *p* < 0.05.

## 3 Results

### 3.1 Patient Characteristics

Of the 11.896 EVT patients in our cohort, 6.093 (51.2%) were female. Women were older (mean age 76.3 ± 12.7 vs. 70.2 ± 12.9 years, *p* < 0.001, *d* = 0.48) and had greater pre‐stroke disability (median pmRS 0 [IQR 0, 2] vs. 0 [IQR 0, 1], *p* < 0.001, *d* = 0.23). Women also had a higher prevalence of hypertension (79.5% vs. 73.9%, *p* < 0.001, *V* = 0.14), AF (46.4% vs. 34.6%. *p* < 0.001, *V* = 0.25), and were less likely to be (current/previous) smoker (16.5% vs. 33.6%, *p* < 0.001, *V* = 0.41). Additionally, women presented with higher baseline NIHSS (median 15 [IQR 9, 19] vs. 14 [IQR 9, 18], *p* < 0.001, *d* = 0.07), indicating more severe strokes at admission.

Cardioembolism was more common in women (55.7% vs. 41.9%, *p* < 0.001, *V* = 0.26), while large‐artery atherosclerosis was more frequent in men (21.5% vs. 32.5%, *p* < 0.001, *V* = 0.23).

Women were less likely to receive IVT (46.9% vs. 49.0%, *p* = 0.019, *V* = 0.04). Successful reperfusion rates were slightly lower in women (83.3% vs. 84.6%, *p* = 0.030, *V* = 0.04). Periprocedural times and complications did not differ significantly between sexes, except for a difference regarding the time from admission to flow restoration (162 [108, 245] vs. 164 [113, 252] min, *p* = 0.027, *d* = 0.05).

**FIGURE 2:**
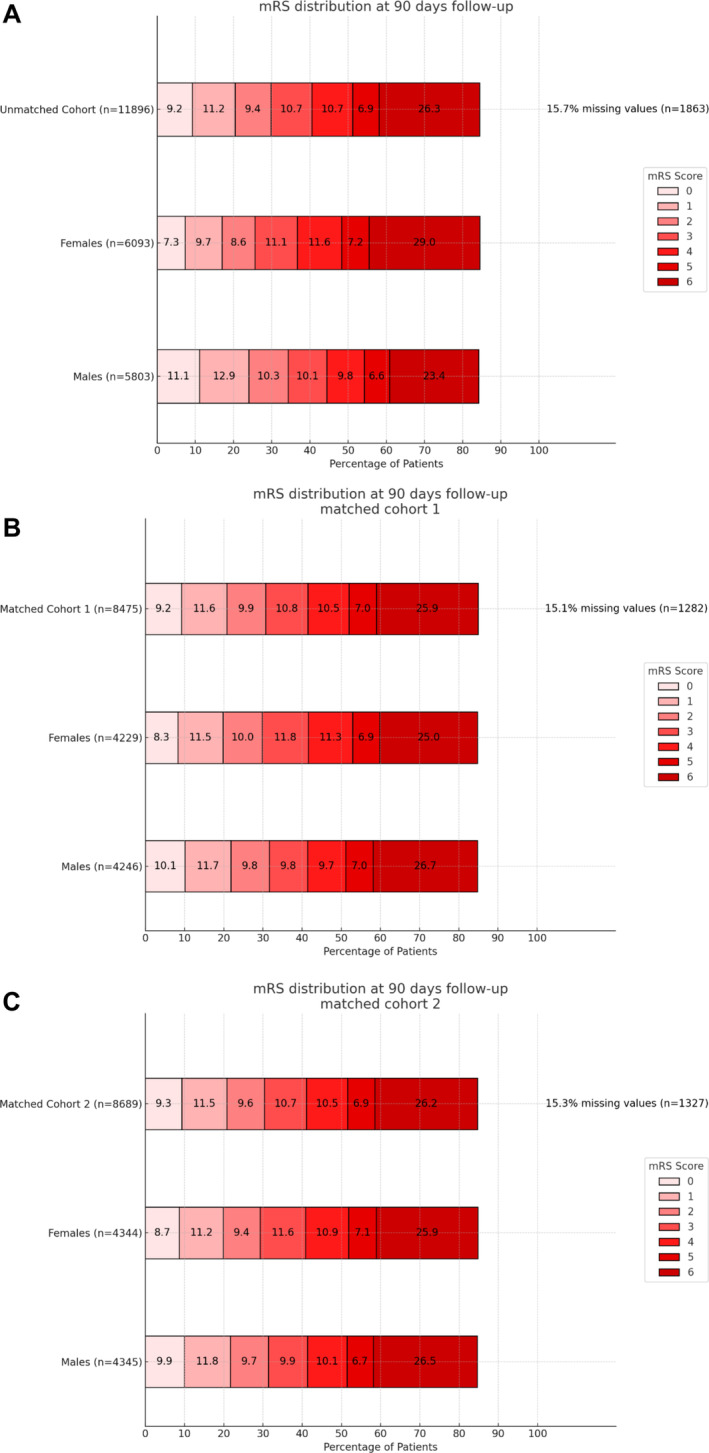
Functional outcome at follow‐up. (A) Unmatched cohort, (B) Cohort 1, (C) Cohort 2. mRS, modified Rankin Scale.

Differences were also observed in outcomes: women had a higher mRS at discharge (median 4 [IQR 2.5] vs. 4 [IQR 2.5], *p* < 0.001, *d* = 0.11) and follow‐up (median 4 [IQR 2.6] vs. 3 [IQR 1.6], *p* < 0.001, *d* = 0.22). Mortality both at discharge (19.0% vs. 15.0%, *p* < 0.001, *V* = 0.11) and follow‐up (29.0% vs. 23.4%, *p* < 0.001, *V* = 0.14) was higher in women. Women had lower rates of good (26.2% vs. 29.1%, *p* < 0.001, *V* = 0.07) and excellent outcomes (14.4% vs. 16.6%, *p* < 0.001, *V* = 0.06) at discharge, as well as at follow‐up (good outcome: 25.5% vs. 34.3%, *p* < 0.001, *V* = 0.21; excellent outcome: 17.0% vs. 24.0%, *p* < 0.001, *V* = 0.20). For details see Table 1 and Figure 2A.

**TABLE 1:** Baseline: Periprocedural and outcome characteristics of males and females in the GSR‐ET (unmatched cohort).

	GSR‐ET *n* = 11.896	Female *n* = 6.093 (51.2%)	Male *n* = 5.803 (48.8%)	*p*	Effect size (cohens *d* or cramers *V*)
Age mean ± SD (years)	73.3 ± 13.2	76.3 ± 12.8	70.2 ± 12.9	< 0.001*	−0.48
Comorbidities, *n* (%)					
Hypertension	9091 (76.4)	4843 (79.5)	4248 (73.2)	< 0.001*	−0.14
DM	2596 (21.8)	1288 (21.1)	1308 (22.5)	0.063	0.03
Dyslipidemia	4772 (40.1)	2330 (38.2)	2442 (42.1)	< 0.001*	0.08
AF	4835 (40.6)	2830 (46.4)	2005 (34.6)	< 0.001*	−0.25
Smoker	2358 (24.8)	1005 (16.5)	1953 (33.6)	< 0.001*	0.41
Previous stroke	837 (7.0)	419 (6.9)	418 (7.2)	< 0.001*	0.03
Anticoagulation at admission	2523 (21.2)	1409 (23.1)	1114 (19.2)	< 0.001*	−0.10
Modified CHAD2DS2‐VASc Score	2.6 ± 1.3	2.9 ± 1.2	2.4 ± 1.3	< 0.001*	−0.37
Etiology, *n* (%)				0.060	−0.03
Large‐artery atherosclerosis (embolus/thrombosis)	3193 (26.8)	1309 (21.5)	1884 (32.5)		
Cardioembolism	5823 (48.9)	3392 (55.7)	2431 (41.9)		
Stroke of other determined etiology	672 (5.6)	276 (4.5)	396 (6.8)		
Stroke of undetermined etiology	2208 (18.6)	1116 (18.3)	1092 (18.8)		
Clinical characteristics at admission					
pmRS, median (IQR)	0 (0, 2)	0 (0, 2)	0 (0, 1)	< 0.001*	−0.23
Baseline NIHSS, median (IQR)	14 (9, 19)	15 (9, 19)	14 (9, 18)	< 0.001*	−0.07
Severe stroke (NIHSS > 15), *n* (%)	5076 (42.7)	2684 (44.1)	2392 (41.2)	0.002*	−0.06
Imaging data, *n* (%)					
Anterior circulation	10,581 (88.9)	5545 (91.0)	5036 (86.8)	< 0.001*	−0.13
Posterior circulation	1330 (11.2)	556 (9.1)	774 (13.3)	< 0.001*	0.13
Treated vessel of occlusion, *n* (%)^a^					
BA	983 (8.2)	413 (6.8)	570 (9.8)	< 0.001*	0.11
VA	256 (2.2)	65 (1.1)	191 (3.3)	< 0.001*	0.15
PCA	384 (3.2)	185 (3.0)	199 (3.4)	0.228	0.02
ACA	365 (3.1)	184 (3.0)	181 (3.1)	0.759	0.01
MCA M1	5967 (50.2)	1295 (21.3)	1132 (19.5)	0.017*	−0.05
MCA M2	2843 (23.9)	1444 (23.7)	1399 (24.1)	0.611	0.01
ICA extracranial	793 (6.7)	298 (4.9)	495 (8.5)	< 0.001*	0.15
ICA intracranial/carotid‐T	2639 (22.2)	1345 (22.0)	1294 (22.3)	< 0.001*	−0.04
Multiple occlusions	1696 (14.3)	760 (12.5)	936 (16.1)	< 0.001*	0.18
Treatment, *n* (%)					
IVT	5703 (47.9)	2857 (46.9)	2846 (49.0)	0.019*	0.04
Successful reperfusion, *n* (%)	9981 (83.9)	5074 (83.3)	4907 (84.6)	0.030*	0.04
Number of passages, mean ± SD	2.1 ± 1.7	2.1 ± 1.7	2.2 ± 1.8	0.087	0.03
Time intervals (min) median (IQR)					
Symptom onset to admission	30 (17, 57)	30 (17, 58)	28 (17, 56)	0.058	−0.046
Door to needle	30 (22, 42)	31 (22, 43)	30 (23, 42)	0.154	0.05
Admission to flow restoration	163 (110, 249)	162 (108, 245)	164 (113, 252)	0.027*	0.05
Symptom onset OR time of recognition of stroke till flow restoration	235 (159, 350)	238 (160, 350)	234 (158, 349)	0.474	−0.02
Periprocedural complications					
Any AE, *n* (%)	2348 (19.7)	1242 (20.4)	1106 (19.1)	0.077	−0.03
sICH, *n* (%)	168 (1.4)	96 (1.6)	72 (1.2)	0.124	−0.03
In‐hospital complications and clinical course					
Recurrent stroke first 24 h, *n* (%)	670 (5.6)	317 (5.2)	253 (6.1)	0.039*	0.04
Any AE during hospital stay, *n* (%)	6840 (57.5)	3509 (57.6)	3331 (57.4)	0.729	−0.01
sICH during hospital stay, *n* (%)	914 (7.7)	475 (7.8)	439 (7.6)	0.590	−0.01
Myocardial infarction during hospital stay, *n* (%)	203 (1.7)	82 (1.3)	121 (2.1)	0.002*	0.06
Hospital stay, mean ± SD (days)	10.6 ± 8.5	10.2 ± 8.0	10.9 ± 9.0	< 0.001*	0.08
Outcome at discharge (*n* = 552 missing values, 4.6%)					
NIHSS, median (IQR)	5 (2, 12)	5 (2, 12)	5 (2, 12)	0.009*	−0.05
Substantial neurological improvement, *n* (%)	5440 (45.7)	2656 (43.6)	2784 (48.0)	< 0.001*	0.08
mRS, median (IQR)	4 (2, 5)	4 (2, 5)	4 (2, 5)	< 0.001*	−0.11
Good outcome, *n* (%)	3285 (27.6)	1596 (26.2)	1689 (29.1)	< 0.001*	0.07
Excellent outcome, *n* (%)	1845 (15.5)	880 (14.4)	965 (16.6)	< 0.001*	0.06
Mortality (mRS 6), *n* (%)	2029 (17.1)	1156 (19.0)	873 (15.0)	< 0.001*	−0.11
Transfer to, *n* (%)				< 0.001*	−0.17
Home, *n* (%)	2576 (21.7)	1181 (19.4)	1395 (24.0)		
Rehabilitation, *n* (%)	5182 (43.6)	2593 (42.6)	2589 (44.6)		
Other hospital, *n* (%)	1800 (15.1)	922 (15.1)	878 (15.1)		
Nursing home, *n* (%)	356 (3.0)	257 (4.2)	99 (1.7)		
Unknown, *n* (%)	1982 (16.7)	1140 (18.7)	842 (14.5)		

Abbreviations: ACA, anterior cerebral artery; AE, adverse event; AF, atrial fibrillation; BA, basilar artery; DM, diabetes mellitus; EVT, endovascular thrombectomy; GSR‐ET, German Stroke Registry‐Endovascular Treatment; ICA, internal carotid artery; IVT, intravenous thrombolysis; MCA, middle cerebral artery; (p)mRS, (premorbid) modified Rankin Scale; NIHSS, National Institutes of Health Stroke Scale; PCA, posterior cerebral artery; sICH, symptomatic intracranial hemorrhage; VA, vertebral artery.

^a^ Multiple occlusions possible.

* Significant.

### 3.2 Univariate Binary Logistic Regression Analyses: Unmatched Cohort

Female sex was consistently associated with worse outcomes compared to males. Females had lower odds of achieving a good outcome both at discharge (OR = 0.63, 95% CI 0.58–0.69, *p* < 0.001) and follow‐up (OR = 0.63, 95% CI 0.58–0.69, *p* < 0.001). Age, higher pre‐stroke disability, and higher NIHSS at admission were also significant predictors of poorer outcomes. Hypertension and diabetes further decreased the likelihood of a good outcome, while smoking surprisingly increased it. Successful reperfusion significantly improved the chances of a good outcome. Regarding mortality, female sex was linked to higher odds both at discharge (OR = 1.32, 95% CI 1.20–1.45, *p* < 0.001) and follow‐up (OR = 1.36, 95% CI 1.25–1.48, *p* < 0.001). Older age, higher pmRS, higher NIHSS, hypertension, and diabetes were all significant predictors of increased mortality. Conversely, smoking was associated with lower mortality. Successful reperfusion substantially reduced the risk of mortality.

### 3.3 Multivariable Binary Logistic Regression: Unmatched Cohort

**FIGURE 3:**
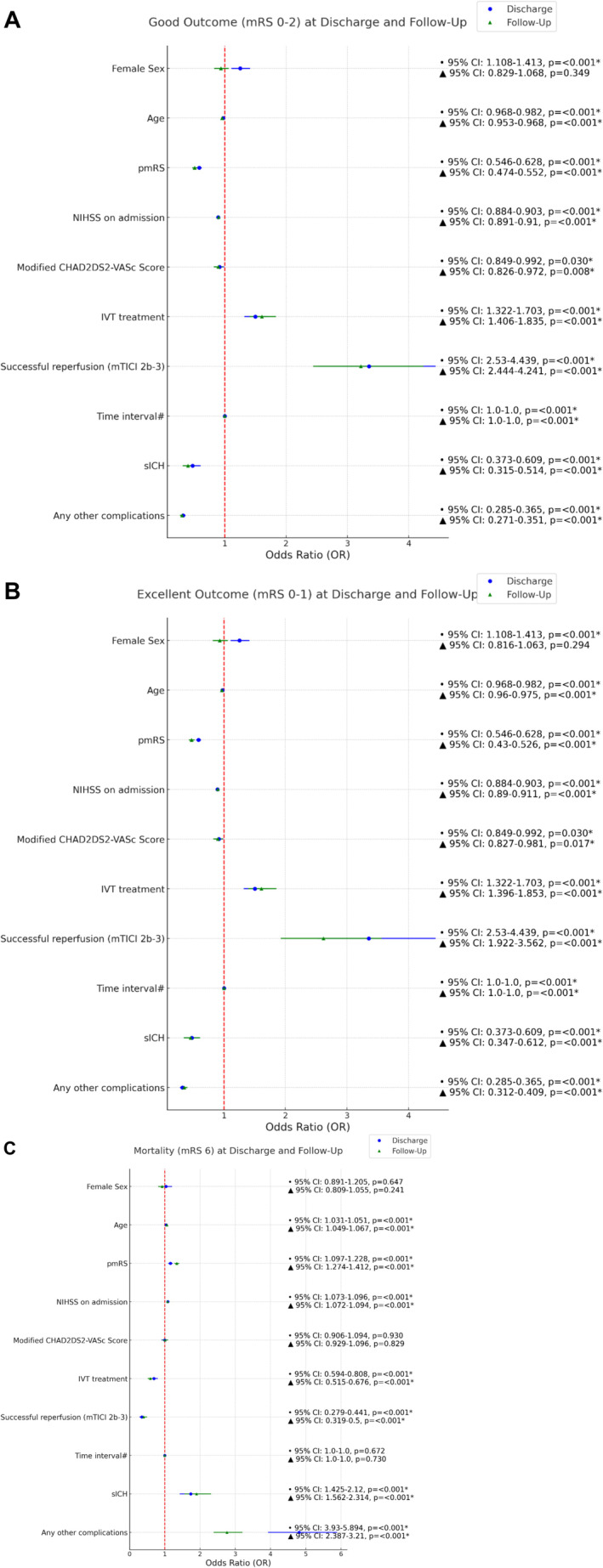
Predictors of outcome at discharge and follow‐up in multivariable binary logistic regression analyses (unmatched cohort). (A) Predictors of good outcome at discharge and follow‐up. (B) Predictors of excellent outcome at discharge and follow‐up. (C) Predictors of mortality at discharge and follow‐up. IVT, intravenous thrombolysis; NIHSS, National Institutes of Health Stroke Scale; (p)mRS, (premorbid) modified Rankin Scale; sICH, symptomatic intracranial hemorrhage. #Time from symptom onset OR time of recognition of stroke until flow restoration [min]. *Significant.

Logistic regression analysis revealed that women had higher odds of achieving good (OR = 1.25, 95% CI: 1.11–1.41, *p* < 0.001) and excellent outcomes at discharge (OR = 1.21, 95% CI: 1.05–1.39, *p* = 0.007) compared to men. Other significant predictors for good outcomes included younger age, lower pmRS, lower NIHSS at admission, IVT treatment, successful reperfusion, absence of sICH, fewer complications, and lower modified CHAD2DS2‐VASc. At follow‐up, female sex was not a significant predictor for good (OR 0.94, 95% CI 0.83–1.07, *p* = 0.349) or excellent (OR 0.93, 95% CI 0.82–1.06, *p* = 0.294) outcomes. For details, see Figure 3A,B. For mortality both at discharge and follow‐up, sex was not a significant predictor (OR = 1.04, 95% CI: 0.89–1.21, *p* = 0.647 and OR 0.92, 95% CI: 0.81–1.06, *p* = 0.241). For details, see Figure 3C.

#### 3.3.1 PSM Cohort 1

The first PSM cohort included 8.475 patients (49.9% women). Women had lower rates of dyslipidemia (38.0% vs. 43.1%, *p* < 0.001, SMD = −0.102), diabetes (20.9% vs. 23.5%, *p* = 0.004, SMD = −0.058), and smoking (19.8% vs. 29.5%, *p* < 0.001, SMD = −0.196), but higher rates of AF (42.5% vs. 39.4%, *p* = 0.005, SMD = 0.062). Subsequently, cardioembolic (51.4% vs. 46.4%, *p* < 0.001) and anterior circulation strokes (90.7% vs. 87.4%, *p* < 0.001, SMD = 0.035) were more common in women. Rates of IVT, completed EVT, and successful reperfusion were similar between sexes. Women experienced more AE at all (20.9% vs. 18.9%, *p* = 0.020, SMD = 0.004), while the incidence of sICH was slightly lower compared to men (6.1% vs. 7.9%, *p* = 0.002). Women had a shorter median time from admission to flow restoration (144 min [IQR 99, 216] vs. 161 min [IQR 112, 249], *p* < 0.001). Both sexes had similar NIHSS at discharge (median 5 [IQR 2, 12] for both, *p* = 0.771), while substantial neurological improvement was slightly higher in women (46.3% vs. 45.8%, *p* = 0.031). Consequently, women had higher odds of good outcomes at discharge (28.5% vs. 26.7%, *p* = 0.022), but lower odds of excellent outcomes at follow‐up (19.8% vs. 21.8%, *p* = 0.021) (Table S1 and Figure 2B).

Univariate analysis showed female sex was associated with higher odds of substantial neurological improvement (OR = 1.10, 95% CI: 1.01–1.21, *p* = 0.031) and good outcome at discharge (OR = 1.12, 95% CI: 1.02–1.23, *p* = 0.022). Conversely, at follow‐up, female sex had lower odds of achieving an excellent outcome (OR = 0.88, 95% CI: 0.79–0.98, *p* = 0.021) (Table S2).

**FIGURE 4:**
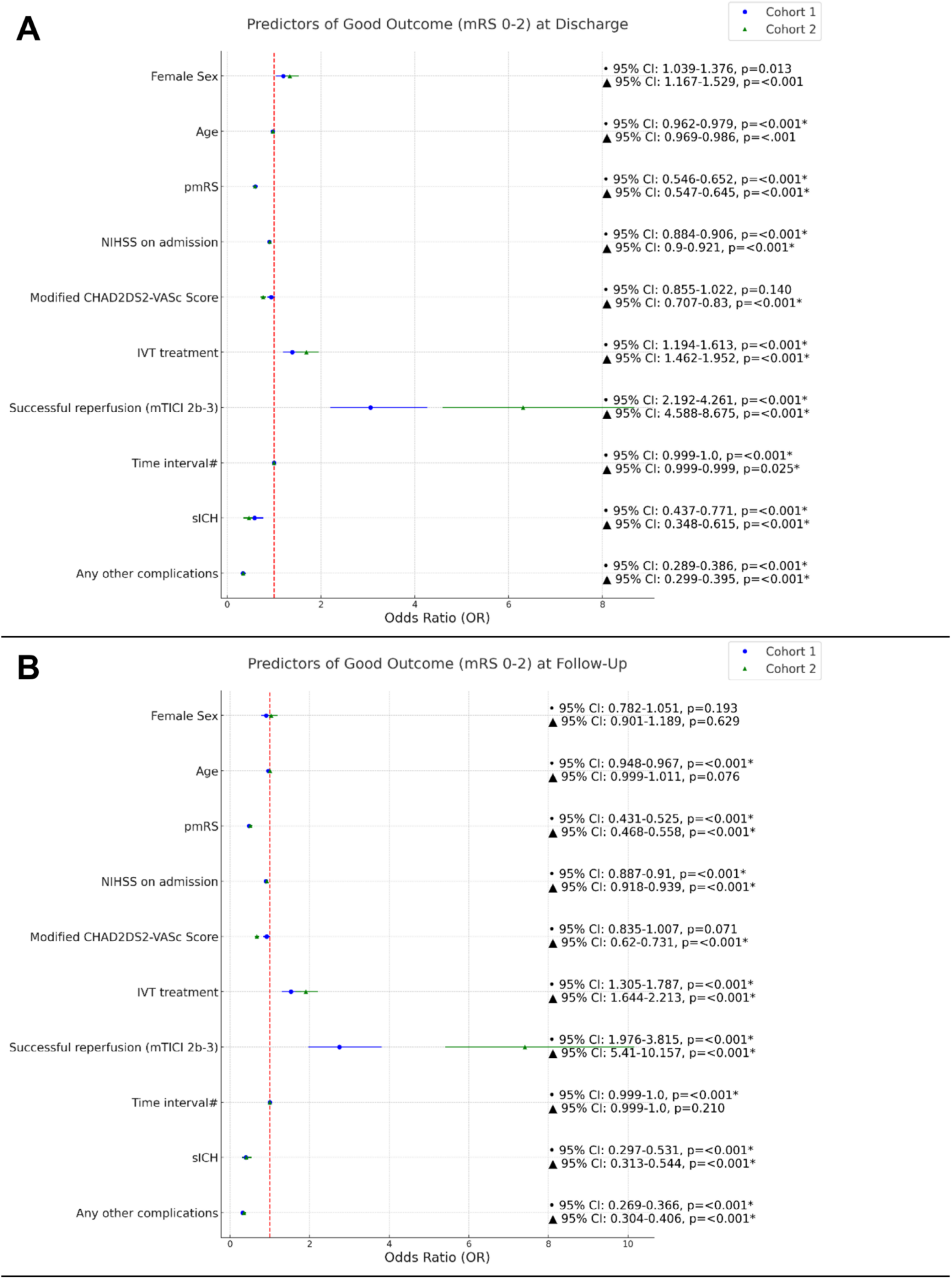
Predictors of outcome at discharge and follow‐up in multivariable binary logistic regression analyses (*matched cohort 1*) and conditional logistic regression (*cohort 2*). (A) Predictors of good outcome at discharge and follow‐up. (B) Predictors of excellent outcome at discharge and follow‐up. (C) Predictors of mortality at discharge and follow‐up. IVT, intravenous thrombolysis; NIHSS, National Institutes of Health Stroke Scale; (p)mRS, (premorbid) modified Rankin Scale; sICH, symptomatic intracranial hemorrhage. #Time from symptom onset OR time of recognition of stroke until flow restoration [min]. *Significant.

Multivariate logistic regression showed higher odds of achieving good (OR = 1.20, 95% CI: 1.04–1.38, *p* = 0.013) and excellent (OR = 1.19, 95% CI: 1.02–1.40, *p* = 0.030) outcomes at discharge for women. At follow‐up, female sex was not a significant predictor for good outcome (OR = 0.91, 95% CI: 0.78–1.05, *p* = 0.193) (Figure 4).

#### 3.3.2 PSM Cohort 2

The second PSM cohort included 8.690 patients (50% women) and was well‐balanced with no significant differences regarding baseline characteristics, stroke severity, and cardiovascular risk factors. Women had lower rates of large‐artery atherosclerosis (24.2% vs. 30.2%, *p* < 0.001, SMD = 0.110) but higher rates of cardioembolic stroke (48.9% vs. 46.1%, *p* = 0.001, SMD = 0.110). IVT administration, EVT completion, and reperfusion rates were similar across sexes, but periprocedural complications were slightly higher in females compared to males (20.6% vs. 18.3%, *p* = 0.006, SMD = 0.056). At discharge, the rates of substantial neurological improvement were similar between sexes (46.6% vs. 45.5%, *p* = 0.202). Good and excellent outcomes at discharge were higher in females compared to males (29.3% vs. 26.6%, *p* = 0.003. and 16.5% vs. 14.7%, *p* = 0.014, respectively). Conversely, at follow‐up, males showed a higher rate of good and excellent outcomes (31.4% vs. 29.4%, *p* = 0.031. and 21.7% vs. 20.0%, *p* = 0.042, respectively). Mortality rates were similar at discharge and follow‐up (17.0% vs. 16.9%, *p* = 0.930. and 25.9% vs. 26.5%, *p* = 0.485. respectively) (Table S3 and Figure 2C).

Univariate analysis revealed that female sex was not significantly associated with complications (OR = 0.97, 95% CI 0.89–1.05, *p* = 0.430) and sICH (OR = 1.10, 95% CI 0.94–1.29, *p* = 0.231). However, it was associated with lower odds of delirium during the hospital stay (OR = 0.56, 95% CI 0.41–0.76, *p* < 0.001). Female sex was significantly associated with higher odds of a good outcome (OR = 1.16, 95% CI 1.05–1.27, *p* = 0.003) and an excellent outcome at discharge (OR = 1.16, 95% CI 1.03–1.30, *p* = 0.014). Similarly, to descriptive analysis, at follow‐up, female sex was associated with lower odds of a good (OR = 0.90, 95% CI 0.82–0.99, *p* = 0.029) and excellent outcome (OR = 0.90, 95% CI 0.81–1.00, *p* = 0.042) (Table S4).

Conditional logistic regression showed that female sex was associated with better outcomes at discharge (OR = 1.34, 95% CI 1.17–1.53, *p* < 0.001 for good outcome; OR = 1.30, 95% CI 1.11–1.52, *p* < 0.001 for excellent outcome), but not at follow‐up (OR = 1.04, 95% CI 0.90–1.19, *p* = 0.629 for good outcome; OR = 1.02, 95% CI 0.88–1.18, *p* = 0.822 for excellent outcome) (Figure 4).

## 4 Discussion

In our large, observational real‐world multicenter study with 11.896 patients from the GSR‐ET, we identified notable results regarding sex‐related differences. The key findings are as follows:

Firstly, in the unmatched cohort, women performed worse in univariate analysis, with lower rates of good and excellent outcomes at both discharge and follow‐up. However, multivariable regression revealed that female sex was associated with higher odds of achieving good and excellent outcomes at discharge, though this advantage did not persist at the 90‐day follow‐up. The shift reflects adjustments for baseline disparities, such as age, pre‐stroke disability, stroke severity, and cardiovascular risk factors, which revealed a protective effect of female sex in short‐term recovery. In the unmatched cohort, women were older, had higher pre‐stroke disability, more hypertension, and AF, and were more likely to have cardioembolic strokes and anterior circulation LVO, while men were more likely to be smokers and had large‐artery atherosclerosis. These findings align with previous research [15, 33, 34, 35, 36, 37, 38, 39]. A systematic review of global sex differences in stroke epidemiology conducted by Appelros et al. found that while stroke incidence was 33% higher in men, women tended to have more severe strokes and higher case‐fatality rates. The prevalence of AF and hypertension was also higher among female stroke patients, potentially contributing to worse outcomes [33]. Mavridis et al. [36] explored more severe strokes in female patients in a study on 2.245 patients aged ≥ 80 years. Reid et al. [15] observed that men were more likely to present with posterior circulation stroke. Despite AF being more prevalent in women and typically associated with anterior circulation strokes, the significant differences persisted even after adjusting for AF, suggesting other underlying factors. Lutsep and Hill [38] examined sex‐related differences in outcomes following EVT in 305 patients using data from the MERCI and Multi MERCI trials. Here, women had higher rates of vertebrobasilar occlusions compared to men. Data from the International Stroke Trial (IST) analyzing 17.370 ischemic stroke patients showed that women faced higher mortality rates at both 14 days and 6 months post‐stroke, and they were more frequently dependent or required institutional care. This study concluded that gender was an independent predictor of death or dependency at 6 months [34].

Women received IVT less frequently than men without differences regarding the time from symptom onset to hospital admission. This disparity persisted even after PSM, suggesting additional factors or decision‐making differences may contribute to the observed treatment gap. Furthermore, while rates of EVT were similar between sexes, women had slightly lower successful reperfusion rates, despite an equal number of EVT passages. Periprocedural complications did not differ. A systematic review by Strong et al. analyzing data from 2008 to 2018 found that women were historically less likely to receive IVT, with 13% lower odds compared to men, though this disparity has lessened over time. Regional variations persisted, with women in the US and most European countries less likely to receive IVT, a trend that was not true in Asia and Germany [13]. European studies showed similar IVT workflow times for both sexes, while North American data indicated slight delays for women [40]. The MR CLEAN Registry study on 3.180 EVT‐patients noted that women had less atherosclerosis but more vessel tortuosity than men, with similar reperfusion rates. Despite these differences, only 34% of women achieved functional independence compared to 46% of men [25]. Mavridis et al. [36] found fewer women received IVT; however, women benefited more from EVT, while men had better outcomes with IVT. A study by Peycheva et al. [37] on 995 anterior circulation LVO patients found that prior vascular events worsened outcomes in both sexes, but men faced more in‐hospital strokes, hemorrhages, and higher mortality at discharge.

To address sex‐specific disparities, we conducted PSM analyses, balancing for age, pre‐stroke disability, stroke severity by NIHSS at admission, and cardiovascular risk factors between sexes. After matching, female sex was consistently associated with better functional outcomes at discharge compared to men in both univariate and multivariate analyses. This highlights the resilience of women in short‐term recovery, even when adjusted. However, this short‐term advantage also did not persist at the 90‐day follow‐up, where men showed higher rates of good and excellent outcomes. Mortality at discharge and follow‐up remained comparable between sexes. The persistence of cardioembolic strokes as a dominant etiology in women, even after PSM, is a significant finding. Cardioembolic strokes are associated with greater stroke severity and higher rates of recurrent embolic events, even with oral anticoagulation, which can adversely affect long‐term outcomes after EVT [41]. Notably, women had significantly more cardioembolic strokes despite being matched for age and cardiovascular risk factors, pointing to the possible role of additional biological or sex‐specific mechanisms, such as variations in clot composition, coagulation pathways, or vascular physiology [42, 43]. Further research is needed to better understand these mechanisms and their impact on stroke outcomes. IVT and EVT rates, as well as successful reperfusion, were consistent between sexes, although women experienced more periprocedural complications.

Our findings are different from earlier studies suggesting that there are no sex‐related differences in EVT outcomes [17, 23, 24, 38, 44, 45, 46]. However, subsequent analyses, including a post hoc analysis from the MR CLEAN trial and studies by Madsen et al., reported worse 90‐day outcomes for women, even after adjusting for age, stroke severity, and pre‐stroke functional status [10, 20]. Wróbel et al. [47] found in a study on 794 patients (51.4% female) that despite smaller early infarct volumes and better cerebral hemodynamics, women had worse functional outcomes. For every 10 mL increase in early infarct volume, females had significantly lower odds of achieving excellent outcomes and higher mortality rates compared to males. Ahnstedt et al. [39] also highlighted significant sex differences in stroke outcomes and treatment responses, with women showing greater benefit from IVT, potentially due to biological differences in clot composition, but this benefit was not uniform across all racial groups. Both sexes benefited equally from EVT. A study by Peycheva et al. [37] on 995 anterior circulation LVO patients found that prior vascular events worsened outcomes in both sexes, but men with prior vascular events had more recurrent in‐hospital strokes and ICH.

Our described disparity—where female sex was associated with better outcomes at discharge, but not at follow‐up—may be influenced by unmeasured factors such as disparities in post‐stroke care, post‐stroke complications, rehabilitation access, or socio‐economic influences. Importantly, female sex was consistently associated with better outcomes at discharge in both the unmatched and matched cohorts, underscoring the significance of short‐term recovery advantages in women after EVT compared to men. While our findings demonstrate this better short‐term recovery, the underlying reasons remain unclear. Neither baseline disparities nor measured clinical factors fully explain this observation, highlighting the need for further research into potential sex‐specific biological and/or hormonal determinants. A cross‐sectional study from the National Health and Nutrition Examination Survey (2013–2018) involving 14.699 adults linked depression to cardiovascular disease (CVD) risks. It found that while women generally had a lower initial risk of cardiovascular disease, those with severe depressive symptoms faced a significantly increased risk compared to men. This highlights the crucial role of integrating mental health support into stroke and cardiovascular disease management, especially for women, to improve long‐term outcomes [48].

The limitations of our study include its observational design without a control group, which limits our ability to directly evaluate the effectiveness of EVT across sexes. Important predictors like infarct volume and collateral status were not included in our data and therefore in the PSM, potentially affecting outcome assessments. Additionally, the presence of missing values and potential selection bias, where EVT might be preferentially given to those expected to benefit most, could influence results. Socioeconomic factors (e.g., educational level, income) were not considered, which may play a critical role in post‐stroke recovery and could explain the loss of short‐term recovery advantages in women. Although our PSM accounted for many confounders, it did not fully explore more complex predictors such as sex‐related differences in post‐stroke depression and other complications. Furthermore, our study was conducted only in Germany, which means that the results may not be applicable to other countries.

In conclusion, our large‐scale study of 11.896 EVT‐treated patients demonstrates that the commonly reported worse outcomes in women cannot be fully explained by baseline differences such as age, pre‐stroke disability, or cardiovascular comorbidities. Conversely, women achieved better short‐term outcomes at discharge, suggesting greater biological resilience in women, even when chronologically older with more cardiovascular risk factors. However, this short‐term advantage was lost during follow‐up, likely due to unmeasured factors such as disparities in post‐stroke care, rehabilitation access, or socio‐economic influences. Our findings underscore the need for personalized medical strategies, further research into sex‐specific biological and hormonal factors in acute care, and tailored long‐term care strategies particularly suited to female patients to address potential socio‐economic challenges affecting women's recovery.

## Author Contributions

**Constanze Single:** formal analysis, data curation, writing – original draft, investigation, visualization, methodology. **Annerose Mengel:** writing – review and editing, resources, conceptualization. **Kornelia Laichinger:** writing – review and editing, resources. **Jennifer Sartor‐Pfeiffer:** resources, writing – review and editing. **Nadja Selo:** resources, writing – review and editing, data curation. **Florian Hennersdorf:** writing – review and editing, resources, data curation. **Benjamin Bender:** writing – review and editing, resources, data curation. **Milani Deb‐Chatterji:** writing – review and editing, validation. **Götz Thomalla:** writing – review and editing. **Joshua Mbroh:** writing – review and editing, data curation, resources. **Sven Poli:** writing – review and editing, data curation. **Ulf Ziemann:** writing – review and editing. **Ulrike Ernemann:** writing – review and editing. **Katharina Feil:** supervision, project administration, conceptualization, writing – original draft, investigation, validation, methodology, visualization.

## Conflicts of Interest

Constanze Single, Kornelia Laichinger, Jennifer Sartor‐Pfeiffer, Nadja Selo, Florian Hennersdorf, Benjamin Bender, Joshua Mbroh, and Ulrike Ernemann are reporting no disclosures. Annerose Mengel received grants from the University of Tübingen (AKF) and speakers' honoraria/consulting fees from AMGEN, all not related to this work. Milani Deb‐Chatterji received research grants from the Werner Otto Stiftung and University Hospital Schleswig‐Holstein and speaker honoraria from Astra Zeneca (not related to this work). Götz Thomalla reports honoraria as consultant or lecturer from Acandis, Alexion, Amarin, Bayer, Boehringer Ingelheim, BristolMyersSquibb/Pfizer, Stryker, and research grants from the European Union (Horizon 2020), German Research Foundation (DFG), and the German Innovation Fund. Sven Poli reports research support from BMS/Pfizer, Boehringer Ingelheim, Daiichi Sankyo, German Federal Joint Committee Innovation Fund, and German Federal Ministry of Education and Research, Helena Laboratories and Werfen as well as speakers' honoraria/consulting fees from Alexion, AstraZeneca, Bayer, Boehringer Ingelheim, Bristol Myers Squibb/Pfizer, Daiichi Sankyo, Portola, and Werfen (not related to this work). Ulf Ziemann received grants from the European Research Council (ERC), German Ministry of Education and Research (BMBF), German Research Foundation (DFG), Else‐Kröner Fresenius Foundation, and consulting fees from CorTec GmbH (not related to this work). Katharina Feil received grants from the University of Tübingen (AKF) and speakers' honoraria/consulting fees from AstraZeneca and BMS/Pfizer (not related to this work).

## Supporting information


Tables S1–S4.

## Data Availability

The data that support the findings of this study are available from the corresponding author upon reasonable request.
