# Supplementary material for: Sex‐Related Differences in Outcomes of Endovascular Treatment in Large Vessel Occlusion Stroke—Analyses From the German Stroke Registry‐Endovascular Treatment
Source: Eur J Neurol. 2025 Feb 26;32(3):e70092. doi: 10.1111/ene.70092 (PMC11863069; doi:10.1111/ene.70092)
Supplement: Supplementary file 1 — Tables S1–S4. [file ENE-32-e70092-s001.docx]

**Supplemental Material**

**Table of Contents**

[Table S1: Baseline, periprocedural and outcome characteristics of females and males in the matched cohort (cohort 1) 2](#_Toc178764247)

[Table S2: Female sex as predictor of outcome parameters in the matched cohort 1 5](#_Toc178764248)

[Table S3: Baseline, periprocedural and outcome characteristics of females and males in the matched cohort (matched cohort 2) 6](#_Toc178764249)

[Table S4: Female sex as predictor of outcome parameters in the matched cohort 2 9](#_Toc178764250)

# Table S1: Baseline, periprocedural and outcome characteristics of females and males in the matched cohort (cohort 1)

|  | **GSR-Population**  **n=8475 (100%)** | **Female**  **n=4229**  **(49.9%)** | **Male**  **n=4246**  **(50.1%)** | **p-value** | **SMD after PSM** |
| --- | --- | --- | --- | --- | --- |
| Age mean±SD (years) | 73.3±12.5 | 73.2±12.5 | 73.3±12.4 | 0.683 | -0.008 |
| **Comorbidities, n (%)** | | | | | |
| Hypertension | 6460 (76.2) | 3231 (76.4) | 3229 (76.0) | 0.943 | 0.004 |
| DM | 1881 (22.2) | 885 (20.9) | 996 (23.5) | 0.004* | -0.058 |
| Dyslipidemia | 3437 (40.6) | 1606 (38.0) | 1831 (43.1) | <0.001* | -0.102 |
| AF | 3470 (40.9) | 1796 (42.5) | 1674 (39.4) | 0.005* | 0.062 |
| Smoker | 2088 (24.6) | 835 (19.8) | 1253 (29.5) | <0.001* | -0.196 |
| Previous stroke | 709 (8.4) | 380 (9.0) | 329 (7.7) | 0.057 | 0.045 |
| Anticoagulation on admission | 1821 (21.5) | 905 (21.4) | 916 (21.6) | 0.811 | 0.000 |
| Modified CHAD2DS2-VASc Score. mean±SD | 2.7±1.3 | 2.7±1.3 | 2.7±1.3 | 0.901 | 0.000 |
| **Etiology, n (%)** | | | | **<0.001*** |  |
| large-artery atherosclerosis (embolus / thrombosis) | 2250 (26.5) | 969 (22.9) | 1281 (30.2) |  | -0.145 |
| cardioembolism | 4143 (48.9) | 2173 (51.4) | 1970 (46.4) |  | 0.101 |
| stroke of other determined etiology (e.g. dissection) | 481 (5.7) | 239 (5.7) | 242 (5.7) |  | 0.000 |
| stroke of undetermined etiology | 1601 (18.9) | 848 (20.1) | 753 (17.7) |  | 0.058 |
| **Clinical characteristics at admission** | | | | | |
| pmRS. median (IQR) | 0 (0. 1) | 0 (0. 1) | 0 (0. 1) | 0.936 | 0.000 |
| pmRS > 2, n (%) | 7225 (85.3) | 3608 (85.3) | 3617 (85.2) | 0.866 | 0.000 |
| baseline NIHSS score. median (IQR) | 14 (9, 18) | 14 (9, 18) | 14 (9, 18) | 0.884 | 0.000 |
| Minor Stroke (NIHSS≤5) | 1002 (11.8) | 510 (12.1) | 492 (11.6) | 0.501 | 0.001 |
| Severe stroke (NIHSS>15), n (%) | 3589 (42.3) | 1757 (41.5) | 1832 (43.1) | 0.136 | 0.001 |
| **Imaging data, n (%)** | | | | | |
| Anterior circulation | 7546 (89.0) | 3836 (90.7) | 3710 (87.4) | <0.001* | 0.035 |
| Posterior circulation | 934 (11.0) | 394 (9.3) | 540 (12.7) | <0.001* | -0.065 |
| **Treated Vessel of occlusion, n (%)^#^** | | | | | |
| BA | 696 (8.2) | 292 (6.9) | 404 (9.5) | <0.001* | -0.090 |
| VA | 169 (2.0) | 56 (1.3) | 113 (2.7) | <0.001* | -0.101 |
| PCA | 280 (3.3) | 129 (3.1) | 151 (3.6) | 0.194 | -0.028 |
| ACA | 266 (3.1) | 141 (3.3) | 125 (2.9) | 0.301 | 0.023 |
| MCA M1 | 4242 (50.1) | 1891 (52.0) | 2044 (48.1) | 0.084 | -0.068 |
| MCA M2 | 2064 (24.4) | 1002 (23.7) | 1062 (25.0) | 0.159 | -0.027 |
| ICA extracranial | 559 (6.6) | 218 (5.2) | 341 (8.0) | <0.001* | -0.113 |
| ICA intracranial/Carotid-T | 1866 (22.0) | 926 (21.9) | 940 (22.2) | 0.124 | -0.007 |
| Multiple occlusions | 1295 (15.3) | 631 (14.9) | 664 (15.6) | <0.001* | -0.018 |
| **Treatment, n (%)** | | | | | |
| IVT | 4023 (47.5) | 1980 (46.8) | 2043 (48.1) | 0.232 | -0.013 |
| Successful reperfusion (mTICI score 2b-3), n (%) | 7146 (84.3) | 3561 (84.2) | 3585 (84.4) | 0.687 |  |
| Number of passages. mean±SD | 2.1±1.8 | 2.1±1.7 | 2.1±1.8 | 0.632 |  |
| General anesthesia during EVT, n (%) | 6216 (73.3) | 3139 (74.2) | 3077 (72.5) | 0.623 |  |
| **Time intervals (minutes). median (IQR)** | | | | | |
| Door to needle | 30 (23, 42) | 31 (23, 42) | 30 (23, 42) | 0.241 |  |
| Admission to flow restoration | 152 (105, 231) | 144 (99, 216) | 161 (112, 249) | <0.001* |  |
| Symptom onset OR time of recognition of stroke till flow restoration | 236 (165, 347) | 244 (171, 350) | 231 (156, 346) | 0.849 |  |
| **Periprocedural complications** | | | | | |
| Any AE, n (%) | 1686 (19.9) | 885 (20.9) | 801 (18.9) | 0.020* |  |
| sICH, n (%) | 101 (1.2) | 50 (1.2) | 51 (1.2) | 0.936 |  |
| **In-hospital complications and clinical course** | | | | | |
| Recurrent stroke first 24 hours, n (%) | 518 (6.1) | 261 (6.2) | 257 (6.1) | 0.793 |  |
| Any AE during hospital stay, n (%) | 4952 (58.4) | 2471 (58.4) | 2481 (58.4) | 0.918 |  |
| sICH during hospital stay, n (%) | 593 (7.0) | 258 (6.1) | 335 (7.9) | 0.002* |  |
| Myocardial infarction during hospital stay, n (%) | 160 (1.9) | 61 (1.4) | 99 (2.3) | 0.003* |  |
| Hospital stay, mean±SD (days) | 10.6±8.7 | 10.4±8.3 | 10.9±9.2 | 0.019* |  |
| NIHSS at 24h follow up. median (IQR) | 10 (4, 18) | 10 (4, 18) | 10 (4, 18) | 0.982 |  |
| **Outcome at discharge (n=422 missing values. 5.0%)** | | | | | |
| NIHSS at discharge, median (IQR) | 5 (2, 12) | 5 (1, 12) | 5 (2, 12) | 0.771 |  |
| Substantial neurological improvement, n (%) | 3906 (46.1) | 1960 (46.3) | 1946 (45.8) | 0.031* |  |
| mRS, median (IQR) | 4 (2, 5) | 4 (2, 5) | 4 (2, 5) | 0.142 |  |
| Good outcome, n (%) | 2341 (27.6) | 1206 (28.5) | 1135 (26.7) | 0.022* |  |
| Excellent outcome, n (%) | 1302 (15.4) | 677 (16.0) | 625 (14.7) | 0.051 |  |
| Mortality (mRS 6), n (%) | 1411 (16.6) | 712 (16.8) | 699 (16.5) | 0.429 |  |

| **Outcome parameters at follow-up (90 days) (n=1282 missing values, 15.1%)** | | | | | |
| --- | --- | --- | --- | --- | --- |
| mRS, median (IQR) | 4 (2, 6) | 4 (2, 6) | 4 (1, 6) | 0.675 |  |
| Good outcome, n (%) | 2603 (30.7) | 1262 (29.8) | 1341 (31.6) | 0.066 |  |
| Excellent outcome, n (%) | 1763 (29.8) | 838 (19.8) | 925 (21.8) | 0.021* |  |
| Mortality, n (%) | 2192 (25.9) | 1059 (25.0) | 1133 (26.7) | 0.070 |  |

ACA: anterior cerebral artery. AE: adverse event. AF: atrial fibrillation, BA: basilar artery. bpm: beats per minute. DM: diabetes mellitus, EVT: endovascular thrombectomy. GSR-ET: German Stroke Registry-Endovascular Treatment. ICA: internal carotid artery. IQR: interquartile range. IVT: intravenous thrombolysis. MCA: middle cerebral artery. (p)mRS: (premorbid) modified Rankin Scale. n: number. NIHSS: National Institutes of Health Stroke Scale. or NIH Stroke Scale. PCA: posterior cerebral artery. sICH: symptomatic intracranial hemorrhage, SD: standard deviation. VA: vertebral artery. #Multiple occlusions possible. *Significant.

# Table S2: Female sex as predictor of outcome parameters in the matched cohort 1

|  | **OR** | **95% CI** | | **p-value** |
| --- | --- | --- | --- | --- |
| sICH | 1.101 | 0.941- | 1.288 | 0.231 |
| Other complication (exluding sICH) | 0.966 | 0.887- | 1.052 | 0.430 |
| delirium | 0.558 | 0.409- | 0.761 | <0.001* |
| Substantial neurological improvement | 1.050 | 0.962- | 1.146 | 0.277 |
| Good outcome at discharge | 1.155 | 1.051- | 1.270 | 0.003* |
| Excellent outcome at discharge | 1.158 | 1.031- | 1.301 | 0.014* |
| Mortality discharge | 1.013 | 0.905- | 1.134 | 0.824 |
| Good outcome at follow-up | 0.899 | 0.818- | 0.989 | 0.029* |
| Excellent outcome at follow-up | 0.896 | 0.806- | 0.996 | 0.042* |
| Mortality at follow-up | 0.961 | 0.870- | 1.061 | 0.427 |

CI: confidence interval. OR: odds ratio. sICH: symptomatic intracranial hemorrhage. *significant.

# Table S3: Baseline, periprocedural and outcome characteristics of females and males in the matched cohort (matched cohort 2)

|  | **GSR-Population**  **n=8690 (100%)** | **Female**  **n=4345**  **(50.0%)** | **Male**  **n=4345**  **(50.0%)** | **p-value** | **SMD after PSM** |
| --- | --- | --- | --- | --- | --- |
| Age mean±SD (years) | 73.0±12.7 | 73.1±13.2 | 72.9±12.1 | 0.090 | 0.013 |
| **Comorbidities, n (%)** | | | | | |
| Hypertension | 6589 (75.8) | 3301 (76.0) | 3288 (75.7) | 0.835 | 0.007 |
| Diabetes mellitus | 1952 (22.5) | 1002 (23.1) | 950 (21.9) | 0.192 | 0.028 |
| Dyslipidemia | 3544 (40.8) | 1785 (41.1) | 1759 (40.5) | 0.429 | 0.012 |
| Atrial fibrillation | 3376 (38.8) | 1657 (38.1) | 1719 (39.6) | 0.111 | 0.030 |
| Smoker | 2047 (23.6) | 974 (22.4) | 1073 (24.7) | 0.080 | 0.053 |
| Anticoagulation on admission | 1791 (20.6) | 860 (19.8) | 931 (21.4) | 0.042* | 0.040 |
| Modified CHAD2DS2-VASc Score. mean±SD | 2.6±1.3 | 2.7±1.3 | 2.6±1.3 | 0.233 | 0.023 |
| **Etiology, n (%)** | | | | **0.001*** | **0.110** |
| large-artery atherosclerosis (embolus / thrombosis) | 2365 (27.2) | 1051 (24.2) | 1314 (30.2) |  |  |
| cardioembolism | 4124 (47.5) | 2124 (48.9) | 2003 (46.1) |  |  |
| stroke of other determined etiology (e.g. dissection) | 515 (5.9) | 259 (6.0) | 256 (5.9) |  |  |
| stroke of undetermined etiology | 1683 (19.4) | 911 (21.0) | 772 (17.8) |  |  |
| **Clinical characteristics at admission** | | | | | |
| pmRS. median (IQR) | 0 (0, 1) | 0 (0, 1) | 0 (0, 1) | 0.344 | 0.021 |
| pmRS > 2, n (%) | 7382 (84.9) | 3709 (85.4) | 3673 (84.5) | 0.223 | 0.023 |
| baseline NIHSS score. median (IQR) | 14 (9. 19) | 14 (9. 19) | 14 (9. 18) | 0.588 | 0.015 |
| Minor Stroke (NIHSS≤5) | 1051 (12.1) | 537 (12.4) | 514 (11.8) | 0.444 | 0.016 |
| Severe stroke (NIHSS>15), n (%) | 3749 (43.1) | 1897 (43.7) | 1852 (42.6) | 0.327 | 0.021 |
| **Imaging data, n (%)** | | | | | |
| Anterior circulation | 7697 (88.6) | 3925 (90.3) | 3772 (86.8) | <0.001* | 0.110 |
| Posterior circulation | 1007 (11.6) | 429 (9.9) | 578 (13.3) | <0.001* | 0.108 |
| **Treated Vessel of occlusion, n (%)^#^** | | | | | |
| BA | 761 (8.8) | 317 (7.3) | 444 (10.2) | <0.001* | 0.104 |
| VA | 183 (2.1) | 56 (1.3) | 127 (3.4) | <0.001* | 0.115 |
| PCA | 290 (3.3) | 141 (3.2) | 149 (3.4) | 0.507 | 0.010 |
| ACA | 263 (3.0) | 132 (3.0) | 131 (3.0) | 0.950 | 0.001 |
| MCA M1 | 4346 (50.0) | 2246 (51.7) | 2100 (48.4) | <0.001* | 0.027 |
| MCA M2 | 1739 (20.0) | 1031 (23.7) | 1053 (24.2) | 0.722 | 0.012 |
| ICA extracranial | 578 (6.7) | 232 (5.3) | 346 (8.0) | <0.001* | 0.106 |
| ICA intracranial/Carotid-T | 1915 (22.1) | 943 (21.7) | 972 (22.3) | 0.025* | 0.048 |
| Multiple occlusions | 1247 (14.3) | 540 (12.4) | 707 (16.3) | <0.001* | 0.173 |
| **Treatment, n (%)** | | | | | |
| IVT | 4174 (48.0) | 2077 (47.8) | 2097 (48.3) | 0.002* | 0.009 |
| Successful reperfusion (mTICI score 2b-3), n (%) | 7312 (84.1) | 3642 (83.8) | 3670 (84.5) | 0.277 | 0.023 |
| Number of passages. mean±SD | 2.1±1.7 | 2.1±1.7 | 2.1±1.8 | 0.699 | 0.001 |
| General anesthesia during EVT, n (%) | 6278 (72.2) | 3146 (72.4) | 3132 (72.1) | 0.305 | 0.017 |
| **Time intervals (minutes). median (IQR)** | | | | | |
| Door to needle | 30 (22, 42) | 31 (22, 43) | 30 (22, 42) | 0.628 | 0.047 |
| Admission to flow restoration | 164 (111, 250) | 163 (109, 248) | 164 (113, 251) | 0.547 | 0.040 |
| Symptom onset OR time of recognition of stroke till flow restoration | 360 (160, 350) | 238 (160, 353) | 235 (160, 348) | 0.753 | 0.017 |
| **Periprocedural complications** | | | | | |
| Any AE, n (%) | 1690 (19.4) | 894 (20.6) | 796 (18.3) | 0.006* | 0.056 |
| sICH, n (%) | 126 (1.4) | 73 (1.7) | 53 (1.2) | 0.070 | 0.038 |
| **In-hospital complications and clinical course** | | | | | |
| Any AE first 24 hours, n (%) | 2733 (31.4) | 1334 (30.7) | 1399 (32.2) |  | 0.056 |
| Recurrent stroke first 24 hours, n (%) | 499 (5.7) | 242 (5.6) | 257 (5.9) | 0.398 | 0.015 |
| Any AE during hospital stay (excluding sICH), n (%) | 4972 (57.2) | 2465 (56.7) | 2507 (57.7) | 0.492 | 0.017 |
| sICH during hospital stay, n (%) | 675 (7.8) | 352 (8.1) | 323 (7.4) | 0.223 | 0.026 |
| Myocardial infarction during hospital stay, n (%) | 152 (1.7) | 66 (1.5) | 86 (2.0) | 0.100 | 0.035 |
| Hospital stay. mean±SD (days) | 10.6±8.5 | 10.4±8.2 | 10.7±8.9 | 0.051 | 0.038 |
| NIHSS at 24h follow up. median (IQR) | 10 (4. 18) | 10 (4. 19) | 10 (4. 18) | 0.590 | 0.005 |
| **Outcome at discharge (n=200 missing values. 4.6%)** | | | | | |
| NIHSS at discharge. median (IQR) | 5 (2, 12) | 4 (2, 5) | 5 (2, 12) | 0.334 | 0.029 |
| Substantial neurological improvement, n (%) | 3999 (46.0) | 2023 (46.6) | 1976 (45.5) | 0.202 | 0.024 |
| mRS. median (IQR) | 4 (2, 5) | 4 (2, 5) | 4 (2, 5) | 0.019* | 0.049 |
| Good outcome, n (%) | 2432 (28.0) | 1275 (29.3) | 1157 (26.6) | 0.003* | 0.066 |
| Excellent outcome, n (%) | 1356 (15.6) | 718 (16.5) | 368 (14.7) | 0.013* | 0.054 |
| Mortality (mRS 6), n (%) | 1476 (17.0) | 740 (17.0) | 736 (16.9) | 0.930 | 0.005 |

| **Outcome parameters at follow-up (90 days) (n=1328 missing values. 15.3%)** | | | | | |
| --- | --- | --- | --- | --- | --- |
| mRS. median (IQR) | 4 (2, 6) | 4 (2, 6) | 4 (1, 6) | 0.278 | 0.023 |
| Good outcome, n (%) | 2639 (30.4) | 1276 (29.4) | 1363 (31.4) | 0.031* | 0.051 |
| Excellent outcome, n (%) | 1809 (20.8) | 868 (20.0) | 941 (21.7) | 0.042* | 0.047 |
| Mortality, n (%) | 2277 (26.2) | 1124 (25.9) | 1153 (26.5) | 0.485 | 0.018 |

ACA: anterior cerebral artery. AE: adverse event. AF: atrial fibrillation, BA: basilar artery. bpm: beats per minute. DM: diabetes mellitus, EVT: endovascular thrombectomy. GSR-ET: German Stroke Registry-Endovascular Treatment. ICA: internal carotid artery. IQR: interquartile range. IVT: intravenous thrombolysis. MCA: middle cerebral artery. (p)mRS: (premorbid) modified Rankin Scale. n: number. NIHSS: National Institutes of Health Stroke Scale. or NIH Stroke Scale. PCA: posterior cerebral artery. sICH: symptomatic intracranial hemorrhage, SD: standard deviation. VA: vertebral artery. #Multiple occlusions possible. Multiple occlusions possible. *significant.

# Table S4: Female sex as predictor of outcome parameters in the matched cohort 2

|  | **OR** | **95% CI** | | **p-value** |
| --- | --- | --- | --- | --- |
| sICH | 1.101 | 0.941- | 1.288 | 0.231 |
| Other complication (exluding sICH) | 0.966 | 0.887- | 1.052 | 0.430 |
| delirium | 0.558 | 0.409- | 0.761 | <0.001 |
| Substantial neurological improvement | 1.050 | 0.962- | 1.146 | 0.277 |
| Good outcome at discharge | 1.155 | 1.051- | 1.270 | 0.003* |
| Excellent outcome at discharge | 1.158 | 1.031- | 1.301 | 0.014* |
| Mortality discharge | 1.013 | 0.905- | 1.134 | 0.824 |
| Good outcome at follow-up | 0.899 | 0.818- | 0.989 | 0.029* |
| Excellent outcome at follow-up | 0.896 | 0.806- | 0.996 | 0.042* |
| Mortality at follow-up | 0.961 | 0.870- | 1.061 | 0.427 |

CI: confidence interval. OR: odds ratio. sICH: symptomatic intracranial hemorrhage. *significant.
